# 酪氨酸激酶抑制剂耐药机制及其治疗策略

**DOI:** 10.3779/j.issn.1009-3419.2011.10.07

**Published:** 2011-10-20

**Affiliations:** 210029 南京，南京市胸科医院呼吸内科 Department of Medical Respiration, Nanjing Chest Hospital, Nanjing 210029, China

人类表皮生长因子受体（epidermal growth factor receptor, EGFR）是原癌基因*C-erbB1*的表达产物，属于酪氨酸激酶生长因子受体家族成员之一，在人类多种实体肿瘤中过度表达，与肿瘤细胞的增殖、侵袭、转移及血管生长等有关^[1, 2]^。选择EGFR为靶点是近年来非小细胞肺癌（non-small cell lung cancer, NSCLC）治疗的前沿手段，第一代表皮生长因子受体酪氨酸激酶抑制剂（EGFR tyrosine kinase inhibitors, EGFR-TKIs）吉非替尼或者厄洛替尼对于复发或者进展期NSCLC显示出良好疗效，但耐药现象的存在是临床面临的一大难题。部分患者存在EGFR-TKIs初始耐药，几乎所有治疗有效的病例在经过一定时间的缓解期后也出现疾病进展。EGFR-TKIs耐药涉及多种机制，本文就目前NSCLC中存在的可能机制及其最新处理策略作一综述。

## 1 *EGFR*突变与EGFR-TKIs疗效的关系

### 1.1 *EGFR*突变与吉非替尼敏感性

*EGFR*突变多发生于基因外显子18、19、20、21，其中与EGFR-TKIs敏感性相关的主要是位于外显子18、21的点突变和外显子19的缺失突变。临床研究^[3-6]^显示外显子19、21突变约占酪氨酸激酶区域突变的90%，含有这些突变可以在一定程度上提高NSCLC对EGFR-TKIs的敏感性，可作为预后良好的预测因子。IPASS研究^[7]^结果显示：对于非吸烟亚裔女性患者，存在*EGFR*突变者是吉非替尼治疗最大获益人群，在突变阳性组中吉非替尼有效率达71.2%，而无*EGFR*突变者有效率仅为1.1%。因此，*EGFR*基因突变检测可以优化治疗方案，预知吉非替尼的临床应用价值。

### 1.2 T790M突变与EGFR-TKIs继发性耐药的关系

T790M是由于EGFR外显子20的第2, 369位的胞嘧啶（C）转化为胸腺嘧啶（T），从而导致第790位的编码产物由苏氨酸替换为甲硫氨酸，已有实验^[6, 8-10]^证实T790M与吉非替尼或者厄洛替尼获得性耐药有关。Onitsuka等^[11]^对10例NSCLC吉非替尼治疗前后的组织样本进行*EGFR*突变分析，结果显示用药前5例为外显子19缺失突变，5例为外显子21点突变，均未发生T790M突变；但在耐药后的组织中则有7例检测到T790M改变，说明T790M突变在吉非替尼继发耐药中属于多发事件。当前理论^[12]^认为：苏氨酸作为“看门人基团”位于酪氨酸激酶接触反应核心之外，与吉非替尼的苯胺基团形成具有高度亲和力的氢键，从而保证了药物与酪氨酸激酶紧密结合而发挥抗肿瘤作用；一旦发生突变，苏氨酸被甲硫氨酸取代，使得该位点上引入了一条更大的氨基酸侧链构成空间位阻，从而影响酪氨酸激酶与吉非替尼之间氢键的形成，最终导致吉非替尼无法与其相结合。

## 2 MET扩增与EGFR-TKIs继发性耐药的关系

人类*MET*基因定位于第7号染色体，其编码产物是肝细胞生长因子（hepatocyte growth factor, HGF）的特异性受体，属于酪氨酸激酶超家族成员之一。多项研究^[8, 13-17]^表明MET扩增与EGFR-TKIs继发性耐药有关，MET通过上调和活化P13K/AKT等途径，直接激活EGFR下游信号通路，导致肿瘤对吉非替尼的耐药。陈志红等^[18]^检测55例术后NSCLC原发病灶和23例EGFR-TKIs耐药肿瘤组织中MET基因扩增情况，结果显示阳性率分别为5.5%（3/55）和21.7%（5/23），两组间有统计学差异；同时比较7例TKIs耐药者治疗前后肿瘤组织中MET的变化情况，发现治疗前均无*MET*基因扩增，治疗后则有2例出现基因扩增，说明MET扩增与EGFR-TKIs继发性耐药有关。

## 3 IGF-1R过表达与EGFR-TKIs继发性耐药的关系

胰岛素样生长因子1受体（insulin-like growth factor-1 receptor, IGF-1R）是一跨膜酪氨酸激酶受体，在许多肿瘤细胞系中过表达，IGF-1R过表达在吉非替尼耐药中也起着特殊的作用^[19, 20]^。EGFR和IGF-1R具有相似的胞外域结构，在IGF-1R过表达的NSCLC细胞中，EGFR-TKIs能诱导EGFR和IGF-1R的异源二聚化作用，激活IGF-1R的下游信号通路，促进肿瘤细胞的增殖。近期一项体外实验^[21]^证实：EGFR-TKIs和IGF-1R抑制剂联用能明显降低靶向受体的磷酸化水平及其下游信号通路蛋白表达，产生明显的肿瘤抑制作用，理论上说明二者联用可作为EGFR-TKIs耐药者新的治疗手段。同样，国内学者联用IGF-1R抑制剂（AG1024）和吉非替尼作用于人NSCLC耐药株，发现二者联用具有较好的协同作用，其抑制细胞增殖和促进细胞凋亡的作用明显增强^[22]^。但目前结论多来源于细胞水平，还需要更多的临床实验去探讨两种抑制剂的最佳组合、最佳治疗人群和药物安全性。

## 4 *K-RAS*基因突变与EGFR-TKIs原发性耐药的关系

*K-RAS*基因在EGFR信号转导通路中起关键作用，基因突变可导致K-RAS的持续激活，具有该突变的患者往往对吉非替尼或厄洛替尼不敏感^[3, 23]^。Mao等^[24]^进行的一项*meta*分析得出：NSCLC中*K-RAS*基因突变率为16%（231/1, 470），腺癌多见，吸烟者较不吸烟者高，突变者吉非替尼客观缓解率为3%（6/210），而野生型者则为26%（287/1, 125）。杨帆等^[25]^构建了肺癌细胞株HCC827（*EGFR*突变、*K-RAS*野生）和H292（*EGFR*、*K-RAS*均野生）进行吉非替尼体外敏感性研究，结果显示：HCC827转染*K-RAS*突变质粒后对吉非替尼的半抑制率（half inhibitory concentration, IC_50_）上升1.8×10^3^倍，敏感性明显下降；H292引入*K-RAS*突变质粒后，IC_50_上升3.0×10^2^倍，敏感性也明显下降，表明无论*EGFR*突变情况如何，出现*K-RAS*基因突变均可引起吉非替尼耐药。

## 5 其它可能的耐药机制

HGF是由机体间质细胞分泌的多功能生物因子，其受体为由原癌基因*MET*编码的具有自主磷酸化活性的跨膜蛋白。HGF与其受体结合后可激活受体酪氨酸蛋白激酶系统，发挥促细胞增殖分化、诱导上皮细胞迁移以及诱发新生血管形成作用。Han等^[26]^对90例入组吉非替尼治疗的患者研究发现：HGF表达水平与吉非替尼反应率明显相关，高表达与吉非替尼耐药有关，可作为一个独立的疗效预测因子。

间变淋巴瘤激酶（anaplastic lymphoma kinase, ALK）属于胰岛素受体酪氨酸激酶超家族成员，在间变性大细胞淋巴瘤中异常活化，目前发现其参与形成的融合基因与多种肿瘤的发生发展相关。在NSCLC中ALK融合基因最常见的融合伴侣是棘皮动物微管结合蛋白4（echinoderm microtubule associated protein-like 4, EML4）。融合基因编码融合蛋白，其导致细胞内ALK酪氨酸激酶区域组成性活化，从而促进细胞的恶性转化。*EML4-ALK*融合基因是NSCLC发生发展过程中独立而又关键的分子靶点，多见于腺癌，*EGFR*、*K-RAS*、*ERBB2*野生型，不吸烟的年轻人群^[27, 28]^，可能是另一潜在的EGFR-TKIs耐药机制。Tiseo等^[29]^在1例48岁非吸烟厄洛替尼二线治疗失败的NSCLC组织中发现了*EML4-ALK*突变。因此，针对这部分患者有必要进行*EML4-ALK*融合基因的检测，并尝试针对ALK的靶向治疗，使不同亚群患者的获益达最大化。

## 6 EGFR-TKIs耐药的处理策略

### 6.1 针对EGFR-TKIs原发性耐药的处理策略

多项临床研究^[3-6]^显示存在*EGFR*基因敏感突变者是靶向药物治疗的最佳获益群体，若不加选择地使用EGFR-TKIs，不仅不能使患者从治疗中获益，反而有可能使*EGFR*野生型者丧失最佳治疗时间。所以，NSCLC患者在接受EGFR-TKIs治疗前必须进行基因突变检测。此外，有研究^[3, 23-24]^表明*K-RAS*基因突变与NSCLC患者EGFR-TKIs原发性耐药有关，如存在基因突变者可对吉非替尼/厄洛替尼的治疗产生抵抗性，针对这些患者可排除使用EGFR-TKIs治疗。因此，临床上对于*EGFR*野生型或者存在*K-RAS*基因突变者，应采用指南推荐的化疗、放疗及其它分子靶点药物为主的综合治疗措施。

### 6.2 针对EGFR-TKIs继发性耐药的处理策略

#### 6.2.1 对于EGFR T790M突变引起的继发性耐药

近年来，EGFR不可逆抑制剂的出现给临床带来曙光，这些药物通过作用于EGFR的ATP结合位点，与受体激酶区域共价结合，且可以同时抑制EGFR受体家族的多个成员。与第一代可逆性EGFR-TKIs相比，不可逆性抑制剂能延长靶点抑制效应，从而增加疗效、减少耐药的发生。EKB-569为最新研制的一种小分子酪氨酸激酶不可逆抑制剂，在体内外可抑制EGFR或者HER2过表达的实体瘤细胞生长，Ⅰ期临床试验发现常见不良事件为腹泻、皮疹、食欲减退，是一种安全有效的口服制剂。Yoshimura等^[30]^对2例存在EGFR突变、对化疗和吉非替尼耐药的晚期NSCLC研究发现，应用EKB-569后显示较好疗效，但目前尚无大规模临床试验证据。PF-00299804能抑制EGFR、HER2、HER4酪氨酸激酶活性，甚至对于T790M突变的NSCLC细胞株也有效，已结束的Ⅰ/Ⅱ期临床研究显示对于晚期NSCLC具有良好的应用前景，目前进一步的临床研究正在进行中。当前尚有多种EGFR不可逆抑制剂被研制出来，比如BIBW-2922、HKI-272（neratinib）、PF-02341066等，少部分已从临床前研究逐渐走向临床，我们拭目以待。

#### 6.2.2 对于c-MET扩增或者IGR-1R过表达引起的继发性耐药

旁路激活途径在EGFR-TKIs耐药中发挥重要作用，针对这些旁路的靶向药物不断涌现。MET抑制剂能在伴有MET过表达的肿瘤细胞中发挥作用，研究^[31]^显示：EGFR-TKIs与MET-TKIs联合，对厄洛替尼耐药（存在L858R/T790M突变）且伴有MET高表达的细胞株有效，但对*EGFR*突变和MET表达均阴性的细胞株无效。P13K/AKT通路是EGFR重要的下游信号通路之一，IGF-1R受体家族能够激活此通路，进而调节肿瘤细胞的代谢、增殖和凋亡，抑制IGF-1R的活性可以抑制肿瘤细胞的生长。实验^[21, 22]^表明IGF-1R抑制剂与EGFR-TKIs联用可增强肿瘤细胞的杀伤作用，因此，针对IGF-1R抑制剂的开发应用成为肿瘤特异性治疗的热点，但真正的临床获益价值还有待于更多的大规模试验去证实。

### 6.3 针对一线EGFR-TKIs治疗耐药后的处理策略

随着多项临床研究结果的公布，包括IPASS、NEJGSG002、WJTOG3405、OPTIMAL等^[7, 32-34]^，EGFR-TKIs在NSCLC一线治疗中的作用进一步得到肯定。NCCN指南推荐如明确有*EGFR*活化突变或者基因扩增的晚期患者，可考虑EGFR-TKI作为其一线治疗选择方案。假如这部分患者出现继发性耐药，其二线治疗目前尚无大规模临床试验及高级别的循证医学证据可供参考。临床上对于体质状态评分较好者仍可选择NCCN指南推荐的化疗方案，但对于体质状态评分较差者则以最佳支持治疗为主（包括中医药治疗）。如果有明确的继发性耐药机制，则可尝试针对性的治疗，如新一代EGFR不可逆性抑制剂、c-MET抑制剂、IGR-1R抑制剂等。

### 6.4 针对二线、三线EGFR-TKIs治疗耐药后的处理策略

晚期NSCLC患者在标准一线化疗方案治疗失败后，NCCN指南推荐体力状态评分较好者可选择EGFR-TKIs作为二线、三线治疗方案，而这些患者在二线、三线治疗失败后NCCN指南推荐需根据患者的一般情况选择不同的治疗策略。如患者体力状态评分为0分-2分，可给予最佳支持治疗或者可参加临床试验；如患者体力状态评分为3分-4分，则仅给予最佳支持治疗。其余可供选择的治疗措施有：继续使用吉非替尼或厄洛替尼似乎仍有一定临床获益，一项回顾性研究^[35]^发现：吉非替尼获得性耐药患者经化疗干预后再次接受吉非替尼治疗仍然有效，可作为晚期NSCLC患者的治疗选择，共有20例患者纳入该实验组，二次用药总的有效率为25%，疾病控制率为65%。另一经常探讨的问题是吉非替尼治疗进展后，换为厄洛替尼是否有效。有研究^[36]^证实：对于吉非替尼治疗失败患者，厄洛替尼的抗肿瘤活性非常有限；但也有资料^[37-39]^认为：虽然厄洛替尼不会作为吉非替尼治疗失败后的常规用药，但部分患者还是可以获益的，主要筛选标准为PS评分较好、吉非替尼初始治疗有效及耐药后的化疗干预。

### 6.5 针对EGFR-TKIs耐药机制不明确者的处理策略

对于这部分患者首先考虑指南推荐的化疗、放疗为主的综合治疗措施，如体力状况评分较差者，则可给予最佳支持治疗（包括中医药治疗）。此外，多靶点酪氨酸激酶抑制剂可以多途径干扰肿瘤细胞的发生、发展，为肺癌EGFR-TKIs耐药患者，特别是晚期患者带来福音。目前应用于临床的药物有索拉非尼、舒尼替尼、凡德他尼等，但大部分多靶点酪氨酸激酶抑制剂仍处于临床试验阶段，其最佳适用人群尚需进一步研究。

## 7 结语

借助于不断进步的现代科学技术手段，人们对于肺癌的治疗已经深入到了分子和基因水平，这为肺癌治疗提供了新的思路与方法。以EGFR为靶点的治疗是当前肺癌治疗的新趋势，但耐药现象的发生使得临床获益明显受限。大量研究表明EGFR-TKIs耐药涉及到多个机制，因此明确不同耐药机制并进行个体化治疗是目前肺癌研究的主要任务。相信经过努力，新一代肺癌靶向治疗药物的开发和临床应用会达到更加成熟的阶段。
